# Innovative pharmacological depression treatment

**DOI:** 10.1007/s00406-025-02040-w

**Published:** 2025-07-31

**Authors:** Hans-Jürgen Möller, Rainer Rupprecht, Andreas Reif

**Affiliations:** 1 Department of Psychiatry and Psychotherapy, University Hospital, LMU Munich, Nussbaumstr. 7, 80336 Munich, Germany; 2 Department of Psychiatry and Psychotherapy, University Hospital, University Regensburg, Universititätsstr.84, 93053 Regensburg, Germany; 3 Department of Psychiatry, Psychosomatic Medicine and Psychotherapy, University Hospital, University Frankfurt, Heinrich- Hoffmann-Str. 10, 60528 Frankfurt am Main, Germany

Treatment of depression with the currently available medications and other neurobiological options is by far not as efficacious as it should be and especially the treatment resistant depression (TRD) needs improved solutions. A recent systematic review and network meta-analysis by Saelens [1] showed very clearly that only six treatments are superior to the other 19, however most of them, apart from ECT, only with a slight advantage. These and other related publications with similar general results demonstrate, that the current common ground of treatment of depression needs urgently innovation and enrichment with new compounds and new mechanisms.

Fortunately the pipeline of new mechanisms and compounds is since the last decade richer than many people in the field might believe. Of course not all new approaches will reach a license or - to expect even more- will be game changers, but at least some of the new developments like especially ketamine/esketamine have already shown their beneficial potential. From there the way to other new developments concerning the glutamatergic pathway might proceed further. But also other mechanisms apart from the currently so potent glutamatergic ones seem promising. Altogether the creativity is remarkable and opens windows in different attractive directions.

In the center of the glutaminergic antidepressants is esketamine, the left turning enantiomer of the racemate ketamine, with its interesting intranasal application mode (FDA license 2019). Especially the ultrarapid efficacy and the efficiency in TRD makes this compound attractive. Together with the racemate ketamine, which, although widely used, however has no license for the treatment of depression. The two compounds form the core group of the so called rapid acting antidepressants (RAADs). The intensified neurobiological research activities on these compounds in the recent years [2, 3] helped to understand the underlying mechanism much better and to understand that beyond the antagonistic acitivity at the glutamatergic NMDA receptor also other neurobiological pathways contribute to the efficacy and rapid onset of action.

Other glutamatergic compounds are also in focus of preclinical or clinical research: Ampakanine (positiv allosteric modulators of the AMPA-receptors), antagonists on the metabotropic glutamate receptors, the NMDA receptor-antagonist dextromethorphan (in combination with bupropion licensed 2022 by the FDA), nitrous oxide as noncompetitive antagonist at the NMDA-receptor [4] etc.

Other mechanism beyond the glutamatergic system are also of interest, like neurostereoids with positive allosteric GABA-A-modulating effects, e.g. zuranolon (2023 licensed by the FDA in the indication post partum depression) and brexanolon [5] From the group of opioidergic compounds partialagonists of the my-opioid- receptor and antagonists at the kappa- opioid receptor (z.B. Navacaprant, Aticaprant) are under investigation [6]. TRPC 4/5 inhibitors lead to anxiolytic and antidepressant effects in mice and show promising results in humans [7]. Orexine receptor antagonists are licensed to treat patients who suffer from insomnia, but they might be also of interest to treat depression.

There is a long tradition of phyto-psychopharmaka. The developments in this field are often not seen as so spectacular as in the other fields of psychopharmacology, because their development is mostly in the hands of smaller family companies. But undoubtedly they are of importance under clinical aspects. Silexan, a lavender extract, showing good results in patients with anxiety disorders, is currently evaluated in depression [8].

Since some years there is a hype for psychedelica in the treatment of depression, powerfully supported by the mass media. Psylocybin (a 5HT-2 A receptor partial -antagonist) seems in the center of this development. The very few adequate studies seem to support the hypothesis, that even a very short treatment with psylocybin is efficacious to treat depression. Up to now a license for the treatment of depression is missing. Further candidates of this group are DMT, 5-MeO-DMT, LSD.

A great hope is that pharmacogenetic research will help to improve the prediction of response /remission and, going beyond this, to predict the optimal medication for the individual patient (precision medicine). However sofar the respective genetic results are not sufficiently satisfying [9]) It might be still necessary to include other neurobiological like e.g. immunological markers [10] or G protein rafts.

This volume brings a selection a papers which demonstrates the vibrant spectrum of research activities in this field. The space of this volume is too restricted to bring all interesting developments, but the selected papers give a hint into important approaches, which might be of future relevance.
